# Using fuzzy logic and mathematical models to predict the financial efficiency of industrial enterprises

**DOI:** 10.1371/journal.pone.0334721

**Published:** 2025-10-30

**Authors:** Yanyan Dong

**Affiliations:** Physical Education College, Henan Sport University, Zhengzhou, Henan, China; Maulana Abul Kalam Azad University of Technology West Bengal, INDIA

## Abstract

The frequent development and unpredictable, dynamic nature of industrial enterprises require an effective financial efficiency detection process. The prediction process uses a large volume of information to identify the details of resources and operational performance in industrial applications. Traditional statistical techniques like regression analysis, decision tree, and machine learning approaches significantly improve prediction efficiency. However, the existing methods face uncertainty, robustness, and scalability issues when exploring high-dimensional data. The research difficulties are addressed by integrating the Fuzzy logic and mathematical model called the FuzzyMath approach. The FuzzyMath concept understands the industrial economic details and predicts financial performance with maximum recognition accuracy. The collected inputs are explored using fuzzy systems that use the multivariate and salp optimization algorithm at every step to improve the overall system efficiency. The optimized membership function, fuzzy rule, and defuzzification process minimize the computation difficulties and can handle the uncertainty issues effectively. Thus, the FuzzyMath-based created rules ensure 99.23% accuracy while predicting financial efficiency in industrial applications.

## 1. Background analysis

Forecasting financial performance efficiency [1,2] is essential in the industrial sector as it serves as a basis for understanding what decisions and strategies to employ. If the financial efficiency forecast done accurately, forecasts allow the organization to understand how resources can be better utilized in operational activities to earn profits [3]. The industries can manage risks, reduce losses, and seize opportunities by finding ineffective areas and predicting future trends [4]. This finanacial data analyzing skill is vital today when the market constantly evolves, and new challenges require quick action and planning. Manufacturing, logistics, and energy sectors can use these insights to optimize their processes and effectiveness [5]. Similarly, forecasting financial performance [6] efficiency improves economic efficiency by optimizing resource allocations [7] and increasing resistance to economic tremors. These capabilities were enhanced due to technological advances, especially in artificial intelligence [8,9] and fuzzy logic systems [10]. As industries become increasingly dependent on data for decision-making, the ability to predict financial performance becomes a requirement for both the development and maintenance of the company. Therefore, effective financial analysis [11,12] improves business robustness, minimizes complexity, and improves competitive edges and confidence while introducing innovations in enterprises.

The above discussions clearly show the importance of finance efficiency prediction. The main issue involved in the forecasting is the volatility and uncertainty [13] attached to financial data, which is always subject to changing markets, forecasting global economies, and unanticipated geopolitical pandemics. The other issue is data quality [14] because error prediction will be standard for these situations simply due to poor data quality, which is either incomplete, inconsistent, or noisy. The third issue is interconnected and nonlinear relationships [15] involving financial variables, making it harder to develop sound models. So, the current landscape has a mix of issues, including real-world complexity, lack of computation power [16,17], and expertise, whereas traditional statical approaches are utilized to reduce these issues. In addition, econometrics fully presents the real systems affected by market, social, and other uncontrollable forces, which are also hard to forecast in particular detail. The absence of metrics generally accepted throughout the sectors also adds to validation, modelling, and benchmarking challenges. Then, ethical issues such as data bias and the opacity of prediction algorithms also add more hurdles. The research difficulties are addressed by introducing fuzzy logic systems that can handle the uncertainty issues. The fuzzy logic system works with multivariate regression analysis and salp optimization algorithms that generate effective fuzzy rules. The generated rules only have relevant rules that directly influence the overall financial prediction efficiency.

Innovative methods are needed to improve financial data prediction accuracy due to its complexity and uncertainty. Traditional models struggle with nonlinear interactions and inadequate data, producing incorrect findings. Fuzzy logic can tolerate uncertainty and record complicated relationships, making it a possible option. This research uses fuzzy logic, multivariate regression, and optimization algorithms to improve financial efficiency prediction in unpredictable financial contexts.

Then, the system’s effectiveness is evaluated using the Python libraries’ experimental results. The general aim of the work is listed as follows.

- To analyze the nonlinearity and uncertainty issues using fuzzy logic that explores the relationship between the variables, ensuring highly reliable and accurate financial data predictions.
- To develop the robust and adaptable FuzzyMath framework by integrating multivariate regression and an optimization algorithm to maintain the inconsistent and incomplete data analysis.
- To evaluate the FuzzyMath system efficiency using the adaptive rule mechanism and external factors like inflation rate and market trends to improve the accuracy of financial efficiency prediction.

FuzzyMath addresses financial prediction problems with a unique combination of fuzzy logic, multivariate regression, and adaptive optimization. Adaptive rule systems that account for inflation and market changes improve flexibility and accuracy. FuzzyMath handles inconsistent and partial data far better than conventional approaches, delivering a reliable financial analysis solution that improves financial efficiency prediction.

Then, the rest of the manuscript is formulated as follows: Section 2 discusses the conventional research analysis in financial data. Section 3 explores the working process of FuzzyMath-based financial efficiency detection and the effectiveness of the system discussed in Section 4. The conclusion is given in section 5.

## 2. Conventional research analysis

### 2.1. Impact of fuzzy logic in financial analysis

Yan et al. [18] analyzed chaotic financial systems using the type-3 fuzzy logic with the Lyapunov approach (T2FL). The main intention of this work is to explore chaos dynamics in economic data to understand the positive and negative Lyapunov index. During the analysis, an attractive dimension test is carried out to identify chaos instability. The efficiency of the system was evaluated using the respective case studies. Safari & Ghaemi [19] forecasting financial data using the hybrid neuro-fuzzy bi-long short-term ensemble model (HNF-BLSTM). This study integrates deep learning concepts and neuro-fuzzy logic to explore the volatile stock data. During the analysis, information on Tesla, Amazon, and JPMorgan datasets was utilized to investigate the case studies. The gathered data is analyzed using fuzzy logic that handles the uncertainty and imprecision issues by ensuring the rule-based analysis. Then, the biLSTM network is incorporated with fuzzy logic to explore complex patterns and long-term dependencies. The captured dependency information used to identify the stock prices and the system’s efficiency is evaluated using benchmark analysis to ensure mean deviation error values compared to conventional methods. Zhao et al. [20] created an early warning system during financial crises using the Attention Fuzzy Neural Networks (AFNN). The AFNN approach intends to provide the earlier sign to eliminate the difficulties during the economic crises and improve the decision-making accuracy. The attention mechanism integrated fuzzy logic observes the relationship between the crisis occurrence and financial indicators. According to the relationship, the risks are predicted, and earlier signs are given. The discussed system uses the 820 listed company information gathered in China, and its financial indicators are continuously explored using an effective attention mechanism. Even though the system effectively works, its interoperability should require additional efforts. Luo & Xiong [21] applied a fuzzy control approach to managing the financial efficiency of wastewater treatment in agriculture. This study aims to improve decision-making efficiency by understanding the present and previous financial management problems in enterprises, which helps to support effective decisions. According to the understanding, suitable solutions are given to diminish the economic risk and maximize the financial efficiency during wastewater treatment. Lakshmi & Kumara [22] unified Procedure for order of preference by similarity to an ideal solution with a randomized weighted fuzzy analytical approach to predict the optimized portfolio stock in financial analysis. The system creates robust portfolio stock selection systems in various scenarios by overcoming uncertainty. The stocks are investigated, and ranks are provided to the stock; according to the rank value, the optimized stocks portfolio is selected, ensuring system efficiency and robustness during the sensitive analysis. Gu et al. [23] applied machine learning techniques to assess credit risk in micro-small enterprises. The imbalanced sampling algorithm solves the class imbalance issues during the analysis. The financial key factors are extracted with the help of XGBoosting features, which help identify low credibility. Then, the SMOTE algorithm is incorporated with boosing features to ensure trust issues and improve the overall credit risk assessment efficiency.

### 2.2. Mathematical model impact on financial analysis

Sangeetha & Alfia [24] suggested a linear regression model to forecast the financial stock market. The prediction process is improved by incorporating training that provides index values for every stock. The regression analysis identifies the stock values with the 500 index, including volume, high, low, close, and open factors. Yang, H., & Wang [25] predicted financial time-series information by applying the Laplace fuzzy information techniques in short-medium enterprises. The distance metrics and linear operations are initially performed on the 1-d fuzzy number space using the asymmetric Laplace fuzzy granule information. Then, trends are extracted with the help of a membership-weighted kernel line. Finally, a long-short-term memory model is applied to forecast the financial time-series data. The system’s efficiency is evaluated by ensuring a high forecasting rate at 0.1 significant level. Supsermpol et al. [26] recommended a random forest and logistic regression approach to predict the Thailand company’s financial performance during the transition period. The integrated machine learning and the statistical model are used to observe the companies’ capability, which helps identify the economic performance of the stock market. The predictive model determines the resource’s priority and improves the overall decision-making analysis.

Adhikari et.al. [34] ranks Indian states by sustainable women’s empowerment indicators. Under uncertainty, the authors use Multi-Criteria Decision-Making (MCDM) with Generalized Triangular Intuitionistic Fuzzy Numbers (GTIFNs) to handle data ambiguity. A new defuzzification algorithm simplifies ranking by converting GTIFNs into crisp values. Policymakers can evaluate and compare women’s empowerment across states using the study’s paradigm. However, the model’s accuracy depends on the input data’s quality and dependability, and criteria selection may affect results.

The Generalized Hukuhara (GH) derivative approach solves the Economic Production Quantity (EPQ) model under uncertainty in Rahaman et al. [35]. The authors use GH differentiability to solve fuzzy differential equations describing the EPQ system in deterministic and fuzzy models. The methodology incorporates uncertainty in production and inventory factors to calculate optimal production volumes that minimize overall cost. The paper shows how the GH derivative works with fuzzy inventory models. The approach requires sophisticated computations and certain fuzziness, which may restrict its generalizability.

A discrete logistic population model with the Allee effect under uncertainty is studied by Alamin et al. [36]. The authors discretize the continuous system using the forward Euler approach and model environmental uncertainty with fuzzy parameters. The study examines how the Allee threshold affects population dynamics, exposing extinction or survival conditions. Under different uncertainty levels, numerical simulations show the model’s behavior. The technique provides insights into population dynamics in uncertain situations, but fuzzy parameters can considerably affect the results and may not capture all ecological intricacies.

Several researchers use mathematical models to identify the industrial enterprise’s financial efficiency and the few aspects described in Table 1. According to various researchers, different statistical techniques and machine-learning approaches are widely utilized to explore financial data. However, the existing methods face uncertainty and robustness issues while processing extensive data. Therefore, the research difficulties are addressed by integrating the fuzzy logic and mathematical model. The detailed working process of the FuzzyMath approach is described in the below section.

**Table 1:** Summary of related studies.

Author	Components										Firm Performance				Methods
	Firm Rate	Solvency	Debt Ratio	Substance	Direct Cost	Utilization Rate	Maintenance	Profit margin	Funding procured	others	ROE	ROS	ROA	Others	
Iltas¸ and Demirgünes¸ [27]	☒	☑	☑	☑	☒	☒	☒	☒	☒	☑	☒	☒	☑	☒	Linear regression
Nabipour et al. [28]	☒	☒	☒	☒	☒	☒	☒	☒	☒	☑	☒	☒	☒	☑	Decision tree, SVM, Adaboost, ANN, and other ML
Shafique et al. [29]	☑	☒	☒	☒	☒	☒	☑	☒	☒	☑	☑	☒	☒	☒	Linear regression
Gao et al [30]	☑	☒	☒	☒	☒	☒	☒	☒	☑	☑	☑	☑	☑	☑	Linear regression
Khan and lqbal et al. [31]	☑	☑	☑	☒	☒	☒	☒	☒	☒	☑	☒	☒	☑	☒	Discriminate analysis (DA)`
Chiadamrong, N., et al. [32]	☑	☑	☑	☑	☑	☑	☒	☒	☒	☒	☑	☑	☑	☒	Multiple regression (MR)
Kayakus et al. [33]	☑	☑	☑	☑	☑	☑	☒	☒	☒	☒	☑	☑	☑	☒	DA & MR
Proposed Work	☑	☑	☑	☑	☑	☑	☒	☒	☒	☒	☑	☑	☑	☒	FuzzyMath

## 3. FuzzyMath model for predicting financial efficiency

**Fig 1:**
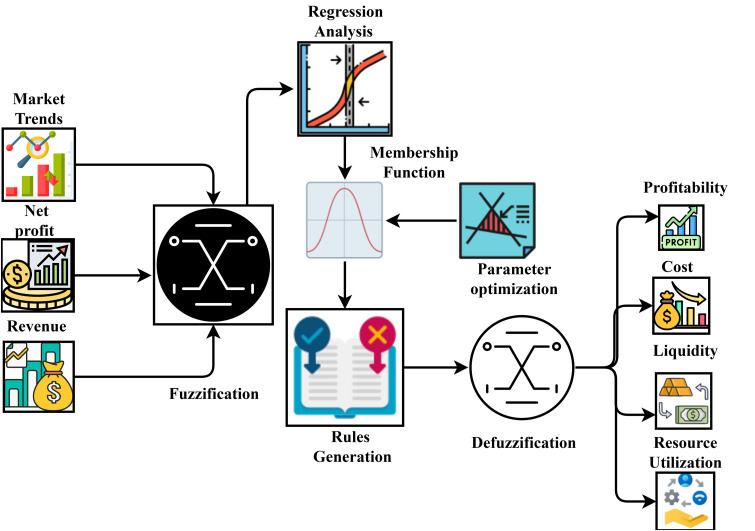
Overall Structure of FuzzyMath-based financial efficiency prediction.

This section discusses the working process for predicting financial efficiency using the FuzzyMath Model. The FuzzyMath model integrates fuzzy logic and mathematical models to explore economic efficiency in industrial enterprises. In this work, multivariate regression analysis and salp optimization model is used in terms of mathematical model to improve the overall prediction efficiency. The introduced model explores financial health by observing the market trends and economic conditions to improve managerial decisions. The FuzzyMath-based approach improves financial efficiency forecast by combining fuzzy logic with optimized regression. Fuzzifying uncertain, imprecise financial data increases interpretability and decision-making. Salp swarm optimization improves fuzzy membership functions and eliminates extraneous rules, boosting prediction accuracy. Adding defuzzification guarantees that complex fuzzy outputs become useful financial insights. The approach is resilient and adaptable using a Company Profit and Expenditures dataset, with 40% designated for testing across data epochs. Because financial markets are dynamic and variable, the technique struggles with real-time data. Future integration of adaptive learning and real-time optimization approaches could support scalability and applicability in global financial systems.The model handles ambiguous issues effectively due to the incorporation of fuzzy logic and the overall working process of financial efficiency analysis shown in Fig 1. The following assumptions and parameter settings are considered to improve the overall prediction efficiency during the analysis.

Fig 1 presents an illustration of a framework for making financial decisions using fuzzy logic. The fuzzy inference system considers various inputs, including market movements, statistical distributions, and even limitations. This system improves decision-making in dynamic financial situations by processing uncertain data and generating outputs such as profit predictions, risk levels, and investment suggestions.

It proves the FuzzyMath-based financial efficiency prediction process’s inclusive working process. The financial inputs $X_{f}$ is processed by the fuzzification process that uses the multivariate regression analysis to identify the relationship between the features. During the study, fuzzification parameters are optimized with the help of the hybridized optimization approach that improves the overall $O_{f}$. The fuzzified inputs are fed into the rule-generation process that predicts the exact $O_{f}$ with minimum prediction error $\left(p_{err}\right)$. Finally, the defuzzification procedure is applied to get the variables used to improve the decision-making efficiency. The $O_{f}$ computation considers the following assumptions, and parameter settings are considered to improve the overall prediction efficiency during the analysis.

### 3.1. Primary assumptions

The FuzzyMath model follows certain assumptions like financial data continuity $\left(d_{c}\right)$, variable interdependence $\left(v_{i}\right)$, the validity of the fuzzy membership function $\left(V_{fm}\right)$ and model optimization $\left(M_{o}\right)$ before predicting the financial efficiency of industries. Financial data continuity assumes typical, smooth data progression without unexpected deviations. This assumption is crucial for forecast accuracy. The model uses financial measurements and controlled random fluctuations to compensate for external interruptions. This method stabilizes prediction even when external variables impact data.Variable interdependence means external, operational, and internal financial issues affect financial efficiency. These components interact and contribute differentially to efficiency. The model weights each element to see how changes in one may affect others. It gives a more complete and realistic view of financial efficiency.

**Assumption 1: Financial data continuity ${(d}_{c})$**

The data $\left(X_{f}\right)$ in smooth trajectory without having any deviation errors except the external factors (Δf interrupt the $X_{f}$. If the Δf disturbs the $X_{f}$ then, the financial metric $F_{m}$ is computed by considering the previous $F_{m-\text{1}}$ and random perturbations (ϵ)

**Assumption 2: Variable Interdependence $(v_{i})$**

The financial efficiency $\left(F_{eff}\right)$ belongs to the Δf, operational (O), financial (F) factors and respective weights $\left(w_{\text{1}},\;w_{\text{2}},\;w_{\text{3}}\right)$.

**Assumption 3: validity of the fuzzy membership function $(V_{fm})$**

Every input in $X_{f}$ is defined with the help of a fuzzy set with an appropriate membership function $\left(\mu_{x}\right)$ to identify the exact output value.

**Assumption 4: model optimization $(M_{o})$**

The parameter in the fuzzy rule and mathematical model is fine-tuned using an optimization approach to reduce the prediction error (err). The $M_{o}$ is done by computing the i^th^ sample-predicted ${\hat{F}}_{eff,i}\;$ and actual $F_{eff}$ for m samples. These assumptions are defined in Equation (1), which helps to improve the $F_{eff}$ accuracy while exploring the large volume of $X_{f}$.


$$\;\begin{array}{c} \begin{array}{c} d_{c}:F_{m}\leftarrow F_{m-\text{1}}+\Delta f+\epsilon \\ v_{i}:F_{eff}\leftarrow w_{\text{1}}.F+w_{\text{2}}O+w_{\text{3}}.\Delta f \end{array}\\ V_{fm}:\sum_{i=\text{1}}^{n}\mu_{i}(x)=\text{1},\;\forall x\\ M_{o}:min\sum_{i=\text{1}}^{m}{\left({\hat{F}}_{eff,i}-F_{eff,i}\right)}^{\text{2}} \end{array}\}$$ (1)

In addition to this Equation (1), input $\left(X_{f}\right)$ and output $\left(O_{f}\right)$ is defined while predicting $F_{eff}$ in industrial enterprises. Then, the parameters are tabulated in Tables 2 and 3.

**Table 2:** $X_{f}$ parameter settings.

Parameters	Notation	Range	Fuzzy Set (FS)	μ(x)	Representation
Net profit margin	ρ	(0,100)	$\mu_{low},\;\mu_{medium},\;\mu_{high}$	Trapezoidal	$\mu_{low}(\rho )=\left\{ \begin{array}{c} \text{0}\;\;\;if\;\rho >\;\rho_{max}\;\;\\ \text{1}\;if\;\rho \leq \;\rho_{min}\\ \frac{\rho_{max}-\rho }{\rho_{max}-\rho_{min}}\;if\;\rho_{min}<\rho \leq \rho_{max} \end{array}\right.\;$
Revenue	γ	(0,1M)	$\mu_{low},\;\mu_{medium},\;\mu_{high}$	Triangular	$\mu_{low}(\gamma )=\left\{ \begin{array}{c} \text{0}\;\;\;if\;\gamma >\;\gamma_{mid}\;\;\\ \text{1}\;if\;\gamma \leq \;\gamma_{min}\\ \frac{\gamma_{mid}-\gamma }{\gamma_{mid}-\gamma_{min}}\;if\;\gamma_{min}<\gamma \leq \gamma_{mid} \end{array}\right.\;$
Market trend index	φ	(−1,1)	$\mu_{neg},\mu_{neu},\mu_{pos}$	Sigmoidal	$\mu_{pos\;}(\varphi )=\frac{\text{1}}{\text{1}+e^{-k(\varphi -c)}}$
Production efficiency	δ	(0,100)	$\mu_{low},\;\mu_{medium},\;\mu_{high}$	Gaussian	$\mu_{eff}(\delta )=e^{-\frac{(\delta -c{)}^{\text{2}}}{\text{2}\sigma^{\text{2}}}}$
Inflation Rate	τ	(0,10)	$\mu_{low},\;\mu_{medium},\;\mu_{high}$	Gaussian	$\mu_{eff}(\tau )==\left\{ \begin{array}{c} \text{0}\;\;\;if\;\tau >\;c\;\;\\ \frac{\tau -a}{b-a}\;if\;a<\tau \leq \;c\\ \frac{c-\tau }{c-b}\;if\;b<\tau \leq c \end{array}\right.\;$

**Table 3:** $O_{f}$ parameter settings.

Parameters	Notation	Range	Fuzzy Set (FS)	μ(x)	Representation
Profitability	$E_{p}$	(0,1)	$\mu_{low},\;\mu_{medium},\;\mu_{high}$	Triangular	$\mu_{low}\left(E_{p}\right)=\left\{ \begin{array}{c} \text{0}\;\;\;if\;E_{p}>\;\text{0}.\text{3}\;\;\\ \text{1}\;if\;E_{p}<\text{0}.\text{1}\\ \frac{\text{0}.\text{3}-E_{p}}{\text{0}.\text{3}-\text{0}.\text{1}}\;if\;\text{0}.\text{1}<E_{p}\leq \text{0}.\text{3} \end{array}\right.\;$
Cost	$E_{c}$	(0,1)	$\mu_{ineff},\;\mu_{moder},\;\mu_{eff}$	Trapezoidal	$\mu_{eff}\left(E_{c}\right)=\left\{ \begin{array}{c} \begin{array}{c} \text{0}\;\;\;if\;E_{c}\leq \text{0}.\text{6}\;\;\\ \text{1}\;if\;\text{0}.\text{8}<E_{c}\leq \text{1}\\ \frac{E_{c}-\text{0}.\text{6}}{\text{0}.\text{8}-\text{0}.\text{6}}\;if\;\text{0}.\text{6}<E_{c}\leq \text{0}.\text{8} \end{array}\\ \text{0}\;\;\;E_{c}>\text{1} \end{array}\right.\;$
Liquidity	$E_{l}$	(0,1)	$\mu_{poor},\mu_{avg},\mu_{good}$	Sigmoidal	$\mu_{pos\;}\left(E_{l}\right)=\frac{\text{1}}{\text{1}+e^{\left(-\text{1}\text{0}.\left(E_{l}-\text{0}.\text{5}\right)\right)}}$
Resource utilization	$E_{r}$	(0,1))	$\mu_{neg},\mu_{neu},\mu_{pos}$	Gaussian	$\mu_{neu}\left(E_{r}\right)=e^{-\frac{{\left(E_{r}-\text{0}.\text{5}\right)}^{\text{2}}}{\text{2}{*\text{0}.\text{1}}^{\text{2}}}}$

### 3.2. Fuzzy logic for $X_{f}\;$ analysis

The fuzzy logic uses the basic steps such as fuzzification, rule generation, and Defuzzification, which helps to process the $X_{f}$ to get the $O_{f}$. The fuzzification process converts the $X_{f}\;$ into the fuzzy set (FS) using fuzzy theorem and mathematical principles.

#### 3.2.1. Fuzzification.

**Theorem 1: Membership Function** μ(x)

The input $X_{f}$ is fed into the fuzzification process, utilizes the μ(x) to get the fuzzy set output values. Consider the fuzzy set (A) over X universal set, than the membership function is defined as $\mu_{A}(x);x\in X$. Xϵ(0,1). $\mu_{A}(x):X\to (\text{0},\text{1})$. Consider the input γ in the medium class defined in Equation (2) using triangular μ(x).


$$\mu_{medium}(\gamma \;)=\left\{ \begin{array}{c} \text{0}\;\;\;\;\;\;\;\;\;\;\;\;\;\;\;\;otherwise\\ \frac{\gamma -\gamma_{low}}{\gamma_{medium}-\gamma_{low}}\;if\;\gamma_{low}\leq \gamma \leq \;\gamma_{medium}\\ \frac{\gamma_{high}-\gamma }{\gamma_{high}-\gamma_{medium}}\;if\;\gamma_{medium}<\gamma \leq \gamma_{high} \end{array}\right.\;$$ (2)

The fuzzification process uses theorem 1 to define the $X_{f}$ to map the *FS,* and the theorem applies the $\mu_{A}\;(x)$ to perform the mapping process. The theorem 1 is defined with the conditions $\mu_{A}\;(x)\in (\text{0},\text{1}\forall \;X;\;\left\{ \begin{array}{c} \mu_{A}\;(x)=\text{0};x\notin A\\ \mu_{A}\;(x)=\text{1};x\in A \end{array}\right.\;$ . For every input $X_{f}$ , the $\mu_{A}\;(x)$ is defined as follows.


$$\;\begin{array}{c} \mu_{\gamma }\;(x)=\text{max}\left(\text{0},\text{min}\left(\frac{x-a}{b-a},\frac{c-x}{c-b}\right)\right)\\ \mu_{\rho }(x)=\text{max}\begin{array}{c} \begin{array}{c} \left(\text{0},\text{min}\left(\frac{x-a}{b-a},\text{1},\frac{d-x}{d-c}\right)\right)\\ \mu_{\delta }(x)=e^{-\frac{(x-c{)}^{\text{2}}}{\text{2}\sigma^{\text{2}}}} \end{array}\\ \mu_{\varphi }(x)= \end{array} \end{array}\}$$ (3)

After $x_{f}\to \mu_{A}(x)$, theorem 2 is applied that uses the intersection and union operations to provide the mathematical functions on generated rules depending on expert choice. The fuzzy rule helps to predict the relationship between the $X_{f}$ and $O_{f}$. Then, the parameters of $\mu_{\gamma }\;(x),\mu_{\rho }(x),\;\mu_{\delta }(x),\;\mu_{\varphi }(xand\;\mu_{\tau }(x)$ shown in Table 4.

**Table 4:** $\mu_{A}\;(x)$ parameter.

(a) Input: γ with $\mu_{A}\;(\gamma )$			
*FS*	*Parameters* (a,b,c)		*Range*
$\mu_{low}(\gamma )$	(0,0,50)		[0,50]
$\mu_{medium}(\gamma )$	(25,50,75)		[25,75]
$\mu_{high}(\gamma )$	(50,100,100)		[50,100]
**(b) Input:** ρ **with** $\mu_{A}\;(\rho )$			
***FS***	***Parameters* (a,b,c,d)**		***Range***
$\mu_{very\;low}(\rho )$	(0,0,10,20)		[0,20]
$\mu_{low}(\rho )$	(10,20,30,40)		[10,40]
$\mu_{medium}(\rho )$	(30,40,60,70)		[30,70]
$\mu_{high}(\rho )$	(60,70,100,100)		[60,100]
**(c) Input:** δ **with** $\mu_{A}\;(\delta )$			
***FS***	** Mean (ω) **	** Standard Deviation (σ) **	***Range***
$\mu_{low}(\delta )$	20	5	[0,40]
$\mu_{medium}(\delta )$	50	10	[30,70]
$\mu_{high}(\delta )$	80	5	[60,100]
**(d) Input:** φ **with** $\mu_{A}\;(\varphi )$			
***FS***	***Parameters* (a,b,c)**		***Range***
$\mu_{neg}(\varphi )$	(0,0,50)		[0,40]
$\mu_{neu}(\varphi )$	(30,50,70)		[30,70]
$\mu_{pos}(\varphi )$	(60,100,100)		[60,100]
**(e) Input:** τ **with** $\mu_{A}\;(\tau )$			
***FS***	***Parameters* (a,b,c,d)**		***Range***
$\mu_{low}(\tau )$	(0,0,2,4)		[0,4]
$\mu_{medium}(\tau )$	(3,4,6,7)		[3,7]
$\mu_{high}(\tau )$	(6,8,10,10		[6,10]

For every input γ,ρ,δ,φ and τ in the fuzzification process with the help of the membership function, multivariate analysis and optimization process, which are explained as follows.

A. **Multivariate regression analysis**

Multivariate regression analysis plays an essential role in improving $F_{eff}$ framework by analyzing the different inputs such as γ,ρ,δ,φ and τ. These γ,ρ,δ,φ and τ are highly interrelated that create an impact on the economic outcomes. Therefore, the multivariate regression analysis is utilized to explore the complex relationship between the features to identify the target outputs. The regression model receives the fuzzified output as input to address the vagueness and uncertainty issues. The regression analysis converts the input to quantifiable terms, improving the inputs' robustness and flexibility. Initially, the inputs $X_{f}$ are scaled as $\frac{x-\text{min}\left(x\right)}{\text{max}(x)-\text{min}(x)}$. During the scaling process in x place γ,ρ,δ,φ and τ replaced between 0 and 1. The scaling process makes the input into a uniform format, and the model training is initiated along with the coefficients $\left(\kappa_{\text{1}},\kappa_{\text{2}},\kappa_{\text{3}},\kappa_{\text{4}},\kappa_{\text{5}}\right)$. The $\kappa_{\text{1}\;}to\;\kappa_{\text{5}}$ used to reduce the error terms (ϵ) which is defined in Equation (4).


$$\;\begin{array}{c} F_{eff}=\kappa_{\text{0}}+\kappa_{\text{1}}\gamma +\kappa_{\text{2}}\rho +\kappa_{\text{3}}\delta +\kappa_{\text{4}}\varphi +\kappa_{\text{5}}\tau +\epsilon \\ \epsilon =\sum_{i=\text{1}}^{n}{\left(F_{eff,i}-\hat{F_{eff,i}}\right)}^{\text{2}}\\ \kappa_{j}^{t+\text{1}}=\kappa_{j}^{t}-\eta \frac{\partial \epsilon }{\partial \kappa_{j}} \end{array}\}$$ (4)

**Fig 2:**
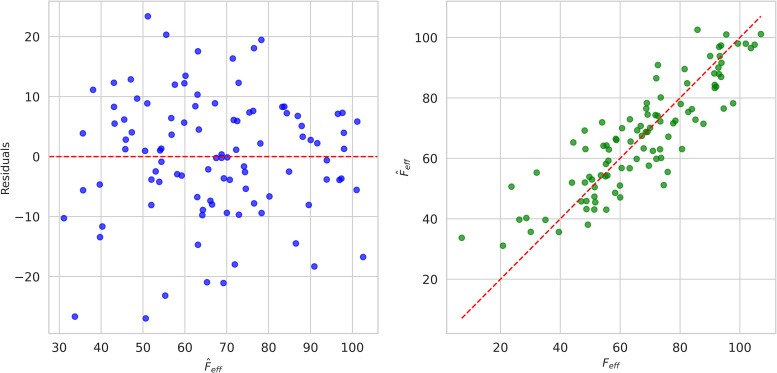
Multivariate analysis of $X_{f}$. **(a)** Residual vs $\hat{F_{eff,i}}$, **(b)** $F_{eff,i}\;vs\;\hat{F_{eff,i}}$.

The multivariate analysis computes the $F_{eff}$ with the help of the intercept $\;{(\kappa }_{\text{0}})$, coefficients $\left(\kappa_{\text{1}},\kappa_{\text{2}},\kappa_{\text{3}},\kappa_{\text{4}},\kappa_{\text{5}}\right)$ and error term (ϵ). This training process reduces the ϵ value, which is identified by computing the deviations between the $F_{eff,i}\;and\;\hat{F_{eff,i}}$. The computed ϵ value determines whether the input is fit into the financial efficiency or not. For every computation, the ϵ value is reduced by fine-tuning the parameters with the help of the learning rate and respective coefficient parameters. The training process provides valuable insights into the multivariate regression model. The Fig 2(a) shows that the relationship between the $residual\;and\;F_{eff}$ in which the analysis depicted that the residual had a uniform spread like constant variance. This uniform spread suggests that the model errors exhibit constant variance, also known as homoscedasticity. Such a distribution is desirable in regression analysis as it implies that the predictive model does not systematically overestimate or underestimate across different values, thereby supporting the model’s reliability and validity in interpreting the data.Fig 2(b) shows that the association between the $F_{eff,i}\;and\;\hat{F_{eff,i}}$ most data points are near the reference lines, ensuring good predictive accuracy. Most data points closely align with the reference line, indicating a strong agreement between predicted and observed results. This proximity reflects high predictive accuracy and suggests that the model effectively captures the underlying patterns in the data. The minimal deviation from the line demonstrates the model’s robustness and reliability in forecasting the effective force across various test cases.

**Fig 3:**
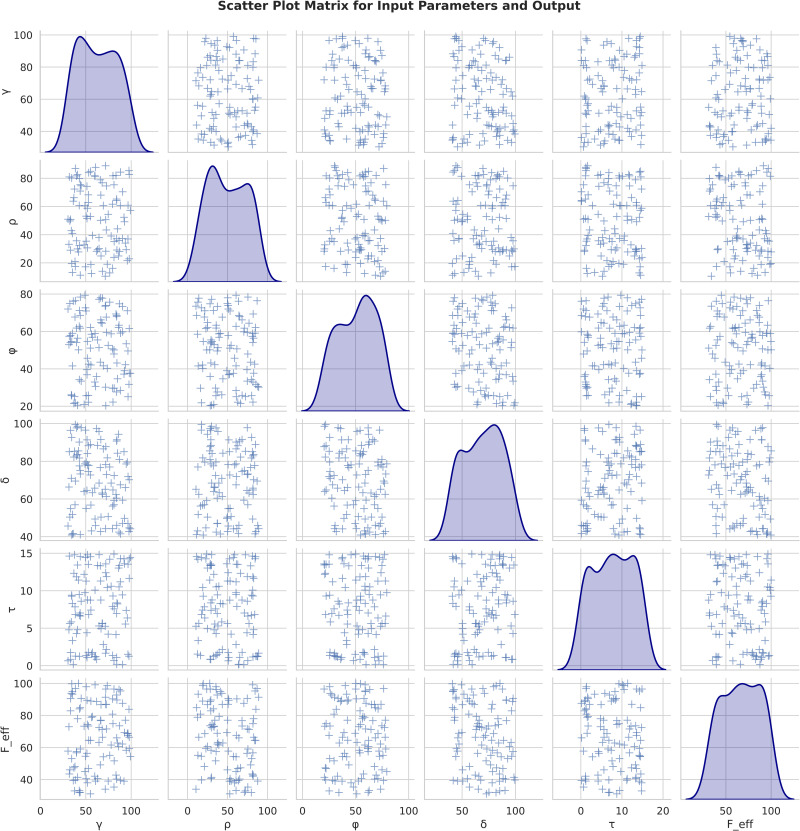
Relationship analysis between $F_{eff,i}\;vs\;X_{f}$.

In addition, the relationship between the γ,ρ,δ,φ and τ vs $F_{eff,i}$ which is explored using the scatter plot analysis (6*6 grid). From the analysis, the diagonal essentials depict that the variable distribution is related to the pair of variables. In this analysis, the γ has the bimodal distribution (20 to 100), ρ has the normal distribution (40 to 50), δ revelations the normal distribution (70 to 80), the φ value centered about 40 defined with right-skewed distribution and τ has 0 to 15 positive skew distribution. These inputs are used to explore the relationship between the inputs and output $f_{eff}\;(\text{4}\text{0}\;to\;\text{1}\text{0}\text{0})$. During the pairwise relationship analysis, few observations of nonlinear relations were indicated as scatter points. The blue points denoted that the observational data represented the significant variability in the observations (Fig 3).

B. **Parameter optimization**

The financial efficiency $\left(F_{eff}\right)$ prediction requires the optimized parameters in fuzzification, rule-based generation, and defuzzification processes. This work applies a salp optimization algorithm to fine-tune the membership function to improve the prediction accuracy. Then, the created rules are evaluated with the help of the optimization approach to reduce the model fitting deviation and eliminate the irrelevant rule participation. Finally, the optimization algorithm improves the defuzzification process to get the exact crisp value from $O_{f}$. Salp optimization works according to its characteristics, which helps fine-tune fuzzy system functions like fuzzification, rule generation, and Defuzzification. The optimization process simplifies the entire process, balances the local and global searching procedure, and ensures flexibility while handling significant constraints and theorems. The Salp population is divided into leaders and followers to provide optimal global solutions. According to the leader’s position and direction, follower fine-tune their position, which helps to find the optimal solution.

**Step 1: Initialization**

In the Salp optimization process, N is the population which has its own position in the search space (ss), and it is defined as $X_{i}=\left(x_{i\text{1}},\;x_{i\text{2}},\;x_{i\text{3}},\ldots \ldots x_{id}\right)$. Here, i is defined as i position, and the dimensionality problem is denoted as d. Along with $X_{i}$, the objective function f(x) is defined to evaluate every $X_{i}$.

**Step 2: Leader** →**Position update**

For every $X_{i}$ its position is updated $x_{i,j}^{t+\text{1}}\;\;$ with the help of $F_{j}$ current best position, lower $x_{ub,j}\;$ and $x_{lb,j}$ boundaries with a coefficient $\left(C_{\text{1}}\right)$ and random numbers $r_{\text{1}}and\;r_{\text{2}}$. Then the $x_{i,j}^{t+\text{1}}\;\;$ process is defined in Equation (5)


$$x_{i,j}^{t+\text{1}}\;\;=\left\{ \begin{array}{c} Fj+C\text{1}.r\text{1}.\;\left(xub,j-xlb,j\right),\;\;\;\;\;\;\;r\text{2}<\text{0}.\text{5}\\ Fj-C\text{1}.r\text{1}.\;\left(xub,j-xlb,j\right),\;\;\;\;\;\;\;r\text{2}\geq \text{0}.\text{5} \end{array}\right.\;$$ (5)

In Equation (5), the $C_{\text{1}}$ value maintains the stability between the exploitation and exploration, which is computed as $\text{2}e^{-\text{4}t/T}$ and $r_{\text{1}}\;and\;r_{\text{2}}$ having value between (0,1).

**Step 3: Follower** →**Position update**

The preceding salps and leaders position, the follower position also updated $x_{i,j}^{t+\text{1}}\;\;$ which help to smoothen the follower movement and it is defined as $x_{i,j}^{t+\text{1}}\;\;=\frac{x_{i-\text{1},j}^{t}\;\;-x_{i,j}^{t}\;\;}{\text{2}}\;,\;i>\text{1}$.

**Step 4: Evaluate** f(x)

Compute the f(x) value for every salp to update the $gb_{sol}$ if a better solution is identified. Repeat the process continuously to reach the ${max}_{itera}$ and expected fitness level f(x). The identified $gb_{sol}$ is considered the best solution. According to the above algorithm steps, the salp optimization algorithm is used to fine-tune the $\mu_{A}\;(x)$ parameters such as spread, peak, and shapes that reduce the prediction error (ϵ). Every slap $\left(X_{i}\right)$ denoted as the potential solution that encoded as a vector consists of parameters (a,b,c or a,b,c, d) which is relevant to the $\mu_{A}\;(x)$. Consider the triangular $\mu_{A}\;(x)=\left\{ \begin{array}{c} \text{0}\;\;otherwise\;\;\\ \frac{x-a}{b-a}\;if\;a\leq x\leq \;b\\ \frac{c-x}{c-b}\;if\;b<x\leq c \end{array}\right.\;$. Each $X_{i}$ fitness f(x) is evaluated to minimize the error value $\epsilon =\frac{\text{1}}{n}\sum_{i=\text{1}}^{n}{\left(y_{i}-{\hat{y}}_{i}\right)}^{\text{2}}$. The error value ϵ is reduced by identifying the optimized parameters based on the salp movement procedure, and the position is updated using Equation (6).


$$x_{i,j}^{t+\text{1}}\;\;=\left\{ \begin{array}{c} F(x)+C\text{1}.r\text{1}.\;\left(xleader-x_{i}^{t}\right),\;\;\;\;\;\;if\;\;i=\text{1}\\ \frac{x_{i-\text{1}}^{t}+x_{i}^{t}}{\text{2}}\;\;\;\;\;\;if\;i>\text{1} \end{array}\right.\;$$ (6)

The updated $x_{i,j}^{t+\text{1}}$ is estimated from control coefficients $C_{\text{1}}$, random number $r_{\text{1}}$, best salp position $x_{leader}$ and fitness function F(x). Based on this process, optimized $\mu_{A}(x)$ is identified that fuzzifies the input value $X_{f}$. Then, the pseudocode for the fuzzification process is shown in Table 5.

**Table 5:** Pseudocode for fuzzification.

Initialization: γ,ρ,δ,φ and τ// input parameter $\mu_{low},\;\mu_{medium},\;\mu_{high}$, $\mu_{pos},\;\mu_{neu},\;\mu_{neg}$ // membership functions $X_{j}$// salp swarm optimization
**Compute** f(x)
**Fuzzification:**
For $\;x\;in\;X_{j}$
Use $\{a,b,c\}\to \mu_{A}$ // defining membership value
Compute $\mu_{A}\;(x)=\left\{ \begin{array}{c} \text{0}\;\;otherwise\;\;\\ \frac{x-a}{b-a}\;if\;a\leq x\leq \;b\\ \frac{c-x}{c-b}\;if\;b<x\leq c \end{array}\right.\;$
**Update** salp position $x_{i,j}^{t+\text{1}}\;$
Leader position update using $x_{i,j}^{t+\text{1}}\;\;=\left\{ \begin{array}{c} Fj+C\text{1}.r\text{1}.\;\left(xub,j-xlb,j\right),\;\;\;\;\;\;\;r\text{2}<\text{0}.\text{5}\\ Fj-C\text{1}.r\text{1}.\;\left(xub,j-xlb,j\right),\;\;\;\;\;\;\;r\text{2}\geq \text{0}.\text{5} \end{array}\right.\;$
Follower update using $x_{i,j}^{t+\text{1}}\;\;=\frac{x_{i-\text{1},j}^{t}\;\;-x_{i,j}^{t}\;\;}{\text{2}}\;,\;i>\text{1}$
**Evaluate** f(x) of $x_{i,j}^{t+\text{1}}\;$
**Recompute** the f(x) of $\;xiafter\;updating\;x_{i,j}^{t+\text{1}}\;$
**Update** $gb_{sol}$
**Repeat** optimization to reach ${max}_{itera}$
**Output:** optimize $\left(\mu_{A}(x)\right)$

The time complexity of the salp swarm optimization algorithm is O(T·n), Where T is the number of iterations and n is the number of salps. It includes constant-time initialization, linear fuzzification, leader position update, follower update, and fitness evaluation steps. Each of these operations scales linearly with the number of salps, and the entire process is repeated for T iterations. This results in an overall linear time complexity, making the algorithm efficient for large populations but dependent on the chosen iteration limit, which directly influences its convergence speed.

#### 3.2.2. Fuzzy rule generation.

The fuzzy rules are engendered by conferring the historical data and expert opinion to identify the relationship between the $X_{f}$ and $O_{f}$. During the rule base generation, theorem 2 is followed to improve the $F_{eff}$ detection process.

**Theorem 2: Fuzzy intersection and union**

The fuzzy intersection and union of FS is defined as minimum and maximum operations. In FS, A and B is defined as two FS, and the intersection theorem and union theorem are defined in Equation (7)


$$\;\begin{array}{c} \mu_{A\cap B}(x)=\text{min}\left(\mu_{A}(x),\;\mu_{B}(x)\right)\\ \mu_{A\cup B}(x)=\text{max}\left(\mu_{A}(x),\;\mu_{B}(x)\right) \end{array}\}$$ (7)

In Equation (7) $\mu_{A\cap B}(x)$ is defined as A and B having minimum membership value and $\mu_{A\cup B}(x)$ has the maximum relationship value between the fuzzy set (FS). The combination of $\mu_{A\cap B}\;\;and\;\mu_{A\cup B}(x)$ is utilized to obtain the fuzzification value of the given input. Let's consider the two inputs $X_{f}:\;\left\{\gamma ,\rho \right\}$ and the membership value of this input is combined to form the rules, which are defined as $\mu_{rule}=min\left(\mu_{high}(\gamma ),\mu_{medium}(\rho )\right)$. These generated rules are aggregated using intersection and union operations to evaluate the rules to predict the $F_{eff}$. The aggregation process is defined with the help of Theorem 3, which is described as follows.

**Theorem 3: Aggregation**

The aggregation theorem is utilized while aggregating multiple FS rules-related outputs. The aggregation process uses the maximum function to get the final output $\mu_{output}\;(x)$. The aggregation is defined using Equation (8)


$$\mu_{output}\;(x)=\text{max}\left(\mu_{rule\text{1}}(x),\mu_{rule\text{2}}(x),\ldots \ldots ..\mu_{rule\text{1}n}(x)\right)$$ (8)

The rules are independent, so outputs are additive while utilizing theorem 3. If the$\mu_{rule\text{1}}(x)$ has 0.5 and $\mu_{rule\text{2}}(x)$ has 0.8, then the aggregation output is $agg_{op}(x)=\text{max}(\text{0}.\text{5},\text{0}.\text{8})=\text{0}.\text{8}$. According to the description, the rules are generated, and the sample rules are shown in Table 6.

**Table 6:** List of generated rules.

No	Rules	Output $(F_{eff})$
1	$If\;\gamma_{high}\;AND\;\rho_{high}\;AND\;\delta_{high}\;$	*High*
2	$If\;\gamma_{medium}\;AND\;\rho_{medium}\;AND\;\delta_{medium}\;\;\;$	*Medium*
3	$If\;\gamma_{low}\;AND\;\rho_{low}\;AND\;\delta_{low}\;\;$	*Low*
4	$If\;\gamma_{high}\;AND\;\rho_{high}\;AND\;\delta_{low}\;\;\;$	*Medium*
5	$If\;\gamma_{medium}\;AND\;\rho_{low}\;AND\;\delta_{high}\;$	*Low*
6	$If\;\gamma_{low}\;AND\;\rho_{medium}\;AND\;\delta_{low}\;$	*Low*
7	$If\;\gamma_{high}\;AND\;\rho_{low}\;AND\;\delta_{high}\;$	*Medium*
8	$If\;\gamma_{medium}\;AND\;\rho_{high}\;AND\;\delta_{medium}\;$	*Medium*
9	$If\;\gamma_{low}\;AND\;\rho_{high}\;AND\;\delta_{high}\;$	*Low*
10	$If\;\gamma_{low}\;AND\;\rho_{low}\;AND\;\delta_{medium}\;$	*Low*
11	$If\;\varphi_{neg}\;AND\;\tau_{high}\;AND\;\gamma_{low\;}AND\;\rho_{low}\;AND\;\delta_{low}\;$	*Low*
12	$If\;\varphi_{neg}\;AND\;\tau_{moderate}\;AND\;\gamma_{medium\;}AND\;\rho_{low}\;AND\;\delta_{medium}\;$	*Low*
13	$If\;\varphi_{stable}\;AND\;\tau_{low}\;AND\;\gamma_{medium\;}AND\;\rho_{medium}\;AND\;\delta_{high}\;$	*Medium*
14	$If\;\varphi_{stable}\;AND\;\tau_{moderate}\;AND\;\gamma_{high\;}AND\;\rho_{high}\;AND\;\delta_{high}\;$	*High*
15	$If\;\varphi_{pos}\;AND\;\tau_{low}\;AND\;\gamma_{high\;}AND\;\rho_{high}\;AND\;\delta_{high}\;$	*High*
16	$If\;\varphi_{pos}\;AND\;\tau_{low}\;AND\;\gamma_{high\;}AND\;\rho_{high}\;AND\;\delta_{high}\;$	*High*
17	$If\;\varphi_{neg}\;AND\;\tau_{low}\;AND\;\gamma_{low\;}AND\;\rho_{medium}\;AND\;\delta_{low}\;$	*Low*
18	$If\;\varphi_{stable}\;AND\;\tau_{high}\;AND\;\gamma_{medium\;}AND\;\rho_{low}\;AND\;\delta_{medium}\;$	*Low*
19	$If\;\varphi_{pos}\;AND\;\tau_{high}\;AND\;\gamma_{high\;}AND\;\rho_{medium}\;AND\;\delta_{high}\;$	*High*
20	$If\;\varphi_{stable}\;AND\;\tau_{low}\;AND\;\gamma_{low\;}AND\;\rho_{medium}\;AND\;\delta_{high}\;$	*Medium*

After forming the aggregated fuzzy rules, multivariate regression and salp optimization are applied to improve the fuzzy system's prediction accuracy, flexibility, and robustness. The computed output is the representation of rule weights $\left(\kappa_{n}\right)$ and membership degree. The multivariate regression analysis computes the relationship between the output and crisp outputs. Then, the regression process is described as $F_{eff}=\kappa_{\text{0}}+\kappa_{\text{1}}R_{\text{1}}+\kappa_{\text{2}}R_{\text{2}}+\kappa_{\text{3}}R_{\text{3}}+\kappa_{\text{4}}R_{\text{4}}\ldots \ldots .\kappa_{n}R_{n}+\epsilon$. Here, the aggregated rules are defined as $R_{n}$, regression coefficients are $\kappa_{n}$ which is used to quantify the fuzzy rule contribution to ensure the output. Then optimize $\left(\mu_{A}(x)\right)$ is applied to get the fuzzified values, which are further pruned with the help of an optimization algorithm to improve the system's interpretability and accuracy. Initially, the rules are encoded and evaluated with the fitness value $f(x)=\frac{\text{1}}{\text{1}+RMSE}$. The computed RMSE value is used to identify the irrelevant rules in the ruleset and optimize the accuracy of rule generation. Then, the contribution of the optimized ruleset in $F_{eff}$is shown in Table 7.

**Table 7:** Optimized fuzzy rules illustration.

#Rule	$agg_{op}\left(x\right)$	$Optimized\;\kappa_{n}$	${Contribution\;(x*\kappa }_{n})$
#R1	0.95	1.26	1.197
#R2	0.89	1.13	1.0057
#R3	0.79	1.05	0.8295
#R4	0.75	1.02	0.765
#R5	0.78	1.23	0.9594
#R6	0.89	1.14	1.0146
#R7	0.93	0.95	0.8835
#R8	0.99	1.02	1.0098
#R9	0.97	1.12	1.0864
#R10	0.96	1.02	0.9792

**Fig 4:**
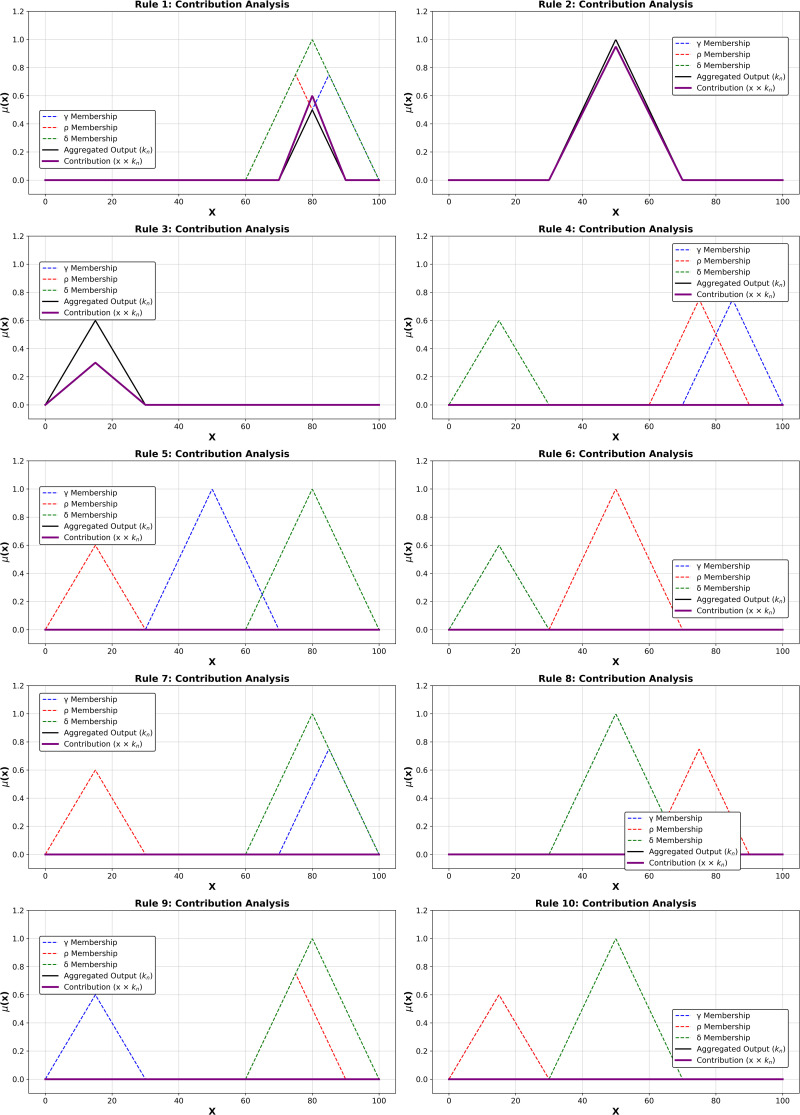
Contribution of aggregated rules in $F_{eff}$.

Table 7 demonstrates that optimized fuzzy rule illustration, which covers the rule index (#Rule) concerning inputs $\gamma ,\rho ,\delta ,\varphi \;and\;\tau ,\;agg_{op}(x)$, $Optimized\;\kappa_{n}$ *and* ${Contribution\;(x*\kappa }_{n})$. From the analysis, rules with higher $agg_{op}(xand\;Optimized\;\kappa_{n}$ contributes high in $F_{eff}$.The optimized parameters highly influence the prediction efficiency and minimize the computation error. In addition, row impact rules are also identified from the list and eliminated throughout the output estimation process. The aggregated rules-related contributions in the output prediction process are shown in Fig 4.

Fig 4 shows fuzzy rule contribution analysis in fuzzy logic. Each subfigure (Rule 1 to Rule 10) shows how y, z, and o membership functions interact in fuzzy inference. Complex fuzzy decision-making is shown by overlapping signals, which represent places where numerous membership functions contribute to the aggregated outcome. Fuzzy logic offers partial membership, allowing a single input to belong to numerous fuzzy sets, boosting flexibility and capturing intricate relationships.

#### 3.2.3. Defuzzification.

The final step is Defuzzification, in which the computed outputs are converted to crisp values to make an effective interpretation. The defuzzification process uses the centroid method to find the output. The defuzzification process uses theorem 4 to get the crisp output value $C_{o}$.

**Theorem 4: Centroid**

The theorem 4 helps to convert the $agg_{out}(x)$ into the crisp value using the centroid approach and the mathematical representation is defined as $C_{o}=\frac{\int x.agg_{out}(x)dx}{\int x.agg_{out}(xdx}$. The $C_{o}$ identifies the area center value under the curve of $\mu_{A}\;(x)$. The $C_{o}$ is derived from the universe discourse variable and aggregated $\mu_{A}(x)$ output value.

According to theorem 4, the defuzzification process is performed, and multivariate regression analysis and optimization techniques are applied to fine-tune the mapping process because the variables are dependent. The defuzzified output values are affected by rule quality and $\mu_{A}(x)$ distribution. The regression analysis computes the associations between the system parameters such as γ,ρ,δ,φ and τ, and $C_{o}$. The relationship is computed as $F_{eff}=\kappa_{\text{0}}+\kappa_{\text{1}}\gamma +\kappa_{\text{2}}\rho +\kappa_{\text{3}}\delta +\kappa_{\text{4}}\varphi +\kappa_{\text{5}}\tau +\epsilon$. Here, the salp swarm optimization is utilized for updating the parameters, such as $\left(\kappa_{\text{1}},\kappa_{\text{2}},\ldots .\kappa_{\text{5}}\right)$. According to the computed position values, the salp algorithm updates the parameters, which minimizes the error values and improves the system’s accuracy and precision rate. Based on the discussions, the obtained output value from Defuzzification is shown in Table 8.

**Table 8:** Defuzzification Output related to $F_{eff}$.

Rules	$F_{eff}$	ρ	γ	φ	δ	τ	$reg\;\left(F_{eff},opt\right)$	Efficiency (%)
**#R1**	95.83	98.4	92.4	94.4	96.35	1.53	96.56	96.44
**#R2**	97.32	93.2	90.6	92.6	95.3	1.22	98.12	97.53
**#R3**	96.14	90.4	88.3	91.3	92.7	1.5	96.84	96.83
**#R4**	98.23	99.2	96.5	97.2	99.2	1.13	99.01	99.23
**#R5**	96.53	94.6	91.3	93.6	92.4	1.35	97.23	97.34
**#R6**	95.95	92.3	89.5	91.3	91.5	1.43	96.45	96.46
**#R7**	97.83	97.3	93.7	94.6	97.3	1.22	98.62	98.74
**#R8**	96.76	93.4	90.3	92.46	93.5	1.14	97.44	97.43
**#R9**	98.42	98.5	95.5	96.3	98.7	1.20	99.15	99.13
**#R10**	97.13	94.3	91.3	93.4	94.2	1.3	97.93	98.4

Table 8 shows that the defuzzification output related to$F_{eff}$ the effective integration of multivariate regression analysis with the salp optimization approach updates the input parameters, ensuring high financial efficiency. From the results, rule 9 and rule 4 ensure high accuracy values such as 99.13% and 99.23%. During the analysis, the regression coefficients clearly understand the relationship between the γ,ρ,δ,φ and τ and $F_{eff}$. The identified relationship highly influences the final financial outcomes. From the analysis, all the formed rules are fine-tuned with the help of multivariate regression with an optimization technique that improves the overall economic outcome prediction accuracy.

## 4. Comparative study analysis

This section discusses the efficiency of the FuzzyMath approach while predicting the financial efficiency. During the analysis Company Profit and Expenditures dataset (https://kaggle.com/datasets/subhamp7/company-profit-and-expenditures) [37] is used to explore the system efficiency. The dataset consists of attributes such as profit, expenditure, marketing-sales information, operational expense, marketing sales, capital expenditures, company revenue, employee details, market shares, financial ratio, years, and industry details. These details are used to analyze the economic efficiency of the industries by applying the FuzzyMath approach. The gathered dataset is divided into testing (40%) and training (60%) sets to evaluate the system’s excellence. The effectiveness of the FuzzyMath system is evaluated using different metrics, such as accuracy $\left(\lambda_{acc}\right)$ (%), efficiency $\left(\lambda_{eff}\right)$ (%), robustness $\left(\lambda_{rob}\right)$, computational time $\left(\lambda_{s}\right)$ and error $\left(\lambda_{err}\right)$. The excellence of the FuzzyMath is compared with existing methods such as the type-3 fuzzy logic with the Lyapunov approach (T2FL) [18], hybrid neuro-fuzzy bi-long short-term ensemble model (HNF-BLSTM) [19], and Attention Fuzzy Neural Networks (AFNN) [20]. The system was developed using Python with respective libraries, and the results obtained are shown in Table 9. In the comparison metrics, the accuracy metric identifies how efficiently the FuzzyMath approach predicts financial metrics like trends, margins, etc. The efficiency metric computes how fast and cost-effectively the FuzzyMath model predicted with maximum accuracy.

**Table 9:** Efficiency analysis of FuzzyMath.

Data Count	Metric	FuzzyMath	T2FL [18]	HNF-BLSTM [19]	AFNN [20]
100	$\lambda_{acc}$	97.8 ± 0.34	94.2 ± 2.3	95.18 ± 0.9	96.15 ± 0.8
	$\lambda_{eff}$	98.9 ± 0.45	95.0 ± 0.18	96.92 ± 0.7	97.08 ± 0.6
	$\lambda_{rob}$	0.97 ± 0.032	0.84 ± 0.023	0.92 ± 0.03	0.902 ± 0.03
	$\lambda_{s}$	12.5 ± 0.78	20.1 ± 1.2	18.35 ± 0.9	16.28 ± 0.8
	$\lambda_{err}$	0.013 ± 0.009	0.041 ± 0.044	0.029 ± 0.003	0.0135 ± 0.002
500	$\lambda_{acc}$	98.0 ± 0.34	94.5 ± 1.3	96.42 ± 0.8	96.19 ± 0.7
	$\lambda_{eff}$	99.0 ± 0.24	95.4 ± 0.5	97.12 ± 0.6	98.12 ± 0.5
	$\lambda_{rob}$	0.98 ± 0.012	0.85 ± 0.023	0.92 ± 0.03	0.934 ± 0.03
	$\lambda_{s}$	13.6 ± 0.88	20.8 ± 1.2	18.39 ± 0.7	17.25 ± 0.8
	$\lambda_{err}$	0.012 ± 0.013	0.040 ± 0.034	0.017 ± 0.002	0.0164 ± 0.002
1000	$\lambda_{acc}$	98.2 ± 0.35	94.7 ± 1.23	96.35 ± 0.7	97.32 ± 0.6
	$\lambda_{eff}$	99.0 ± 0.36	95.6 ± 0.38	97.15 ± 0.5	98.12 ± 0.5
	$\lambda_{rob}$	0.98 ± 0.012	0.85 ± 0.043	0.92 ± 0.02	0.932 ± 0.02
	$\lambda_{s}$	14.8 ± 0.91	21.5 ± 1.23	19.17 ± 0.7	17.43 ± 0.8
	$\lambda_{err}$	0.012 ± 0.007	0.042 ± 0.004	0.022 ± 0.003	0.0167 ± 0.002
5000	$\lambda_{acc}$	98.3 ± 0.52	94.8 ± 1.21	96.37 ± 0.7	97.45 ± 0.6
	$\lambda_{eff}$	99.1 ± 0.36	95.7 ± 0.7	97.17 ± 0.5	98.13 ± 0.5
	$\lambda_{rob}$	0.98 ± s0.03	0.86 ± 0.012	0.93 ± 0.02	0.934 ± 0.02
	$\lambda_{s}$	15.2 ± 1.3	21.9 ± 1.3	19.4 ± 0.8	17.28 ± 0.7
	$\lambda_{err}$	0.011 ± 0.008	0.041 ± 0.044	0.015 ± 0.002	0.0124 ± 0.002
10,000	$\lambda_{acc}$	98.5 ± 0.45	95.0 ± 1.23	96.4 ± 0.6	97.17 ± 0.6
	$\lambda_{eff}$	99.2 ± 0.34	95.8 ± 0.46	97.3 ± 0.5	98.35 ± 0.4
	$\lambda_{rob}$	0.99 ± 0.03	0.86 ± 0.013	0.91 ± 0.02	0.924 ± 0.02
	$\lambda_{s}$	16.3 ± 1.2	22.5 ± 1.45	20.2 ± 0.9	18.15 ± 0.8
	$\lambda_{err}$	0.010 ± 0.007	0.040 ± 0.003	0.015 ± 0.002	0.012 ± 0.002

**Fig 5:**
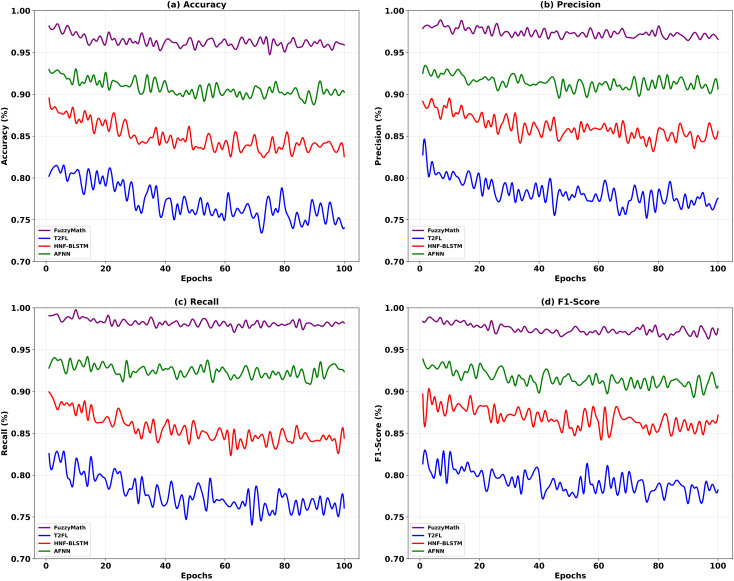
FuzzyMath excellence analysis.

Table 9 illustrates the efficiency analysis of the FuzzyMath compared with existing methods in which the system ensures scalability, robustness, accuracy, and efficiency while predicting financial efficiency. The introduced uses the multivariate regression with optimized based selected fuzzy membership function that converts the input to the fuzzified values. The generated fuzzified values ensure stability while exploring the large and small datasets. During the computation, the error value is computed for every actual and predicted value that identifies the optimized parameters. The selected parameters minimize the error value and improve the overall prediction accuracy. Along with the benchmark analysis, the excellence of the FuzzyMath efficiency is evaluated at different iterations, and the result is shown in Fig 5.

Four models—FuzzyMath, T2FL, HNF-BLSTM, and AFNN—are compared in Fig 5 for accuracy, precision, recall, and F1-score. Over 100 epochs, FuzzyMath exceeds other models in stability and accuracy. Fuzzy-based approaches can handle ambiguity and complex decision limits, demonstrating the benefits of incorporating fuzzy logic into financial efficiency prediction. FuzzyMath is competent in forecasting financial efficiency through its up to several variables regression technique and salp optimization strategies within a fuzzy logic system. It has some predictive capabilities compared to other advanced methods, such as the TT2FL, Hybrid HNF-BLSTM, and AFNN (Fig 5). FuzzyMath models all the relationships between financial variables and facilitates modeling real-life uncertainties by implementing up to several variables regression. Because of salp optimization, tuning the membership functions and the rule parameters becomes dynamic, improving the system and its decision-making processes. The importance of the FuzzyMath approach is that it does not suffer the problems encountered with T2FL, which takes a long time, as they are based on Lyapunov stability theories. FuzzyMath, like HNF-BLSTM, does not overfit when applying deep learning ensembles on small datasets. Also, FuzzyMath achieves better understanding and flexibility through combining optimization strategies than AFNN, especially since AFNN suffers from a clarity problem in attention mechanism. Taking all into consideration, FuzzyMath is the best approach for predicting finance efficiency due to its unique features of combining statistical equations and bio-inspired methods.

## 5. Conclusion

Thus, the paper explains the prediction of FuzzyMath-based financial efficiency by utilizing the predefined input and output parameter settings. The gathered information is processed by a fuzzification process that converts the input into a fuzzy set with different membership functions. The analysis uses multivariate regression and salp optimized fitness value to fine-tune the membership function. Then, fuzzy rules are generated with the help of the fuzzy membership function, which is further updated using the optimization algorithm, reducing the irrelevant fuzzy rules. The irrelevant elimination process improves the overall prediction accuracy. Finally, the optimization algorithm is incorporated with the defuzzification process that converts the fuzzified output into crisp output, showing 99.23% accuracy, and the fuzzy rule is integrated with the fuzzy efficiency analysis. The discussed system uses the Company Profit and Expenditures dataset, in which 40% of data is utilized for testing, ensuring robustness and adaptabiltiy in different epochs and data. Even though the system effectively works, the approach faces difficulties in real-time data. In the future, the model should incorporate learning and optimization techniques to ensure sustainability while analyzing the global market data.The integration of real-time financial data streams, the enhancement of fuzzy rule adaption through deep learning, and the expansion of model applicability across a variety of industrial sectors will be the primary focuses of future study. In addition, hybrid optimization strategies will be investigated in order to further enhance the accuracy of predictions and decision support for decision making in order to maximize financial efficiency in dynamic economic contexts.
